# Supplementary material for: Biocompatible fluorocarbon liquid underlays for in situ extraction of isoprenoids from microbial cultures
Source: RSC Adv. 2022 Jun 6;12(26):16632–9. doi: 10.1039/d2ra01112c (PMC9169902; doi:10.1039/d2ra01112c)
Supplement: RA-012-D2RA01112C-s001 [file RA-012-D2RA01112C-s001.pdf]

Electronic Supplementary Material (ESI) for RSC Advances.
This journal is © The Royal Society of Chemistry 2022

Supporting information for

Biocompatible fluorocarbon liquid underlays for in situ extraction of isoprenoids from microbial cultures

Sebastian Overmans *[a] and Kyle J Lauersen [a]

[a] Biological and Environmental Sciences and Engineering Division, King Abdullah University of Science and Technology (KAUST), Thuwal 23955-6900, Saudi Arabia

Contents of this file

Suppl. Figures 1 & 2

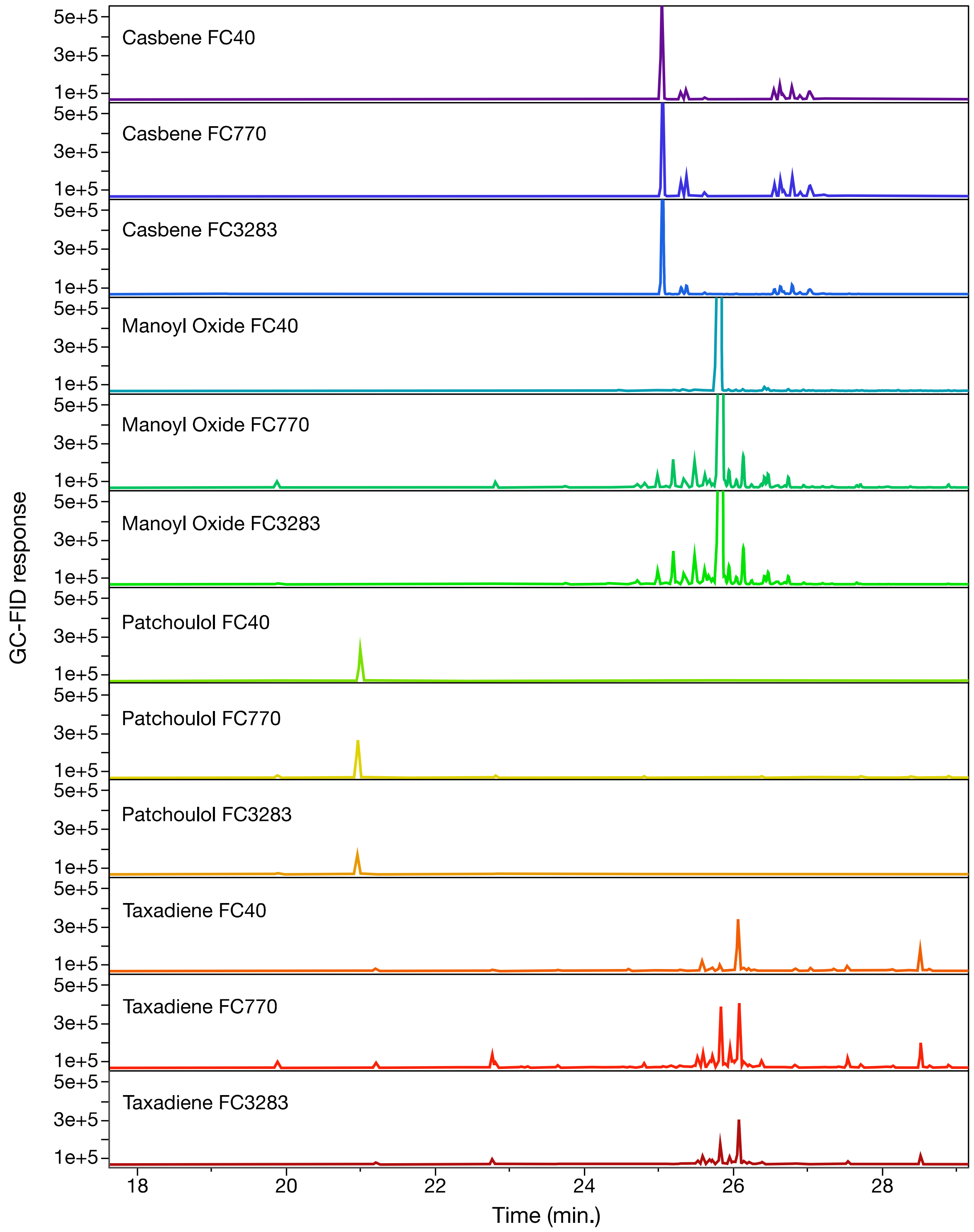

Suppl. Figure 1. Representative GC-FID chromatograms of FC-40, FC-70 and FC-3283 underlays after 10 d of 2-phase solvent-medium culturing with different engineered terpenoid-producing *C. reinhardtii* strains.

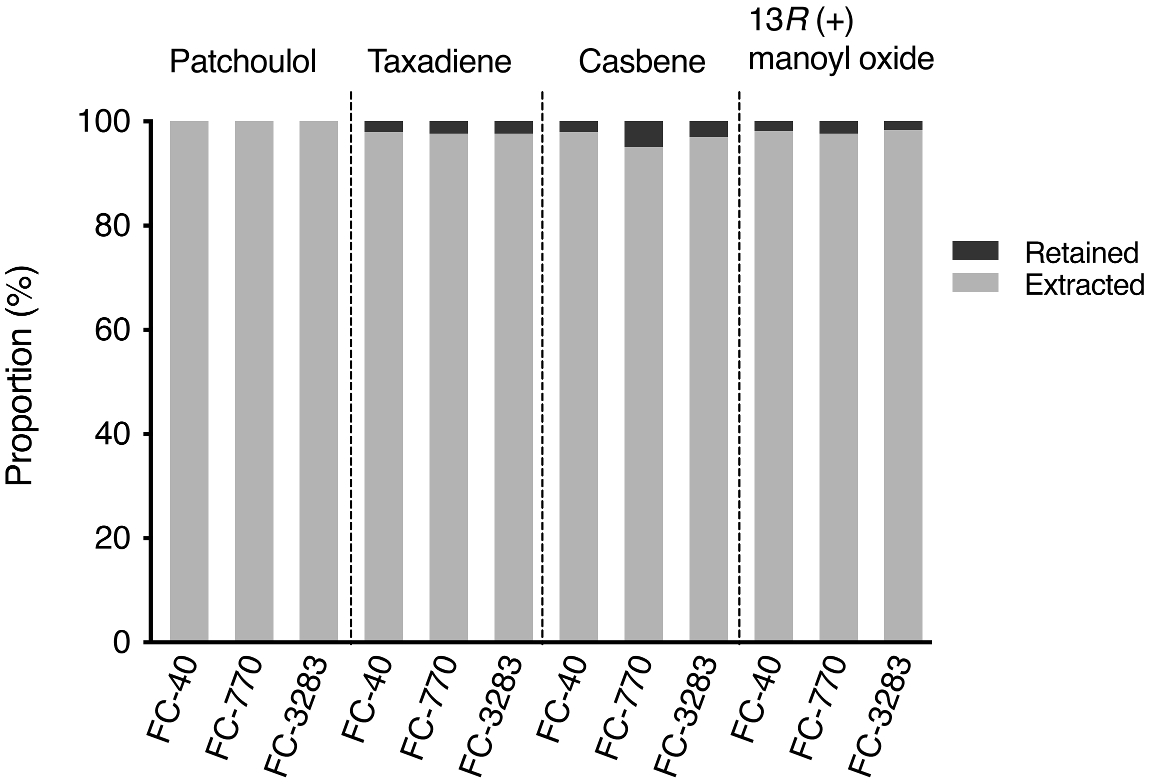

Suppl. Figure 2. Extraction efficiency of various terpenoids accumulated in different FCs after accumulation from two-phase engineered algal culture. Liquid-liquid extraction with 96% ethanol (1:1 v/v) for 16 h was performed on FCs. Grey bars indicate the proportion (%) of terpenoid concentration observed in ethanol after extraction compared to starting concentration in each FC.
